# Profiling and Functional Analyses of MicroRNAs and Their Target Gene Products in Human Uterine Leiomyomas

**DOI:** 10.1371/journal.pone.0012362

**Published:** 2010-08-24

**Authors:** Jiri Zavadil, Huihui Ye, Zhaojian Liu, JingJing Wu, Peng Lee, Eva Hernando, Patricia Soteropoulos, Gokce A. Toruner, Jian-Jun Wei

**Affiliations:** 1 Department of Pathology, School of Medicine, New York University, New York, New York, United States of America; 2 Cancer Institute and Center for Health Informatics and Bioinformatics, New York University, New York, New York, United States of America; 3 Department of Pathology, Feinberg School of Medicine, Northwestern University, Chicago, Illinois, United States of America; 4 Department of Microbiology and Molecular Genetics, Public Health Research Institute, University of Medicine and Dentistry of New Jersey, Newark, New Jersey, United States of America; 5 Department of Pediatrics, Institute of Genomic Medicine, University of Medicine and Dentistry of New Jersey, Newark, New Jersey, United States of America; Roswell Park Cancer Institute, United States of America

## Abstract

**Background:**

Human uterine leiomyomas (ULM) are characterized by dysregulation of a large number of genes and non-coding regulatory microRNAs. In order to identify microRNA::mRNA associations relevant to ULM pathogenesis, we examined global correlation patterns between the altered microRNA expression and the predicted target genes in ULMs and matched myometria.

**Methodology/Principal Findings:**

Patterns of inverse association of microRNA with mRNA expression in ULMs revealed an involvement of multiple candidate pathways, including extensive transcriptional reprogramming, cell proliferation control, MAP kinase, TGF-β, WNT, JAK/STAT signaling, remodeling of cell adhesion, and cell-cell and cell-matrix contacts. We further examined the correlation between the expression of the selected target gene protein products and microRNAs in thirty-six paired sets of leiomyomas and matched myometria. We found that a number of dysregulated microRNAs were inversely correlated with their targets at the protein level. The comparative genomic hybridization (CGH) in eight ULM patients revealed that partially shared deletions of two distinct chromosomal regions might be responsible for loss of cancer–associated microRNA expression and could thus contribute to the ULM pathogenesis via deregulation of target mRNAs. Last, we functionally tested the repressor effects of selected cancer-related microRNAs on their predicted target genes *in vitro*.

**Conclusions/Significance:**

We found that some but not all of the predicted and inversely correlated target genes in ULMs can be directly regulated by microRNAs *in vitro*. Our findings provide a broad overview of molecular events underlying the tumorigenesis of uterine ULMs and identify select genetic and regulatory events that alter microRNA expression and may play important roles in ULM pathobiology by positively regulating tumor growth while maintaining the non-invasive character of ULMs.

## Introduction

Uterine leiomyomas (ULMs) are the most common benign smooth muscle tumors in women of reproductive age. About 40% of ULMs contain non-random chromosomal anomalies involving a small number of specific chromosomal regions [1]. Global gene expression profiling of ULMs revealed that hundreds of genes are dysregulated including those with functional roles in cell proliferation, differentiation and extracellular matrix production [2]. So far, only a few specific genes or cytogenetic aberrations have been identified to be associated with ULMs. While many of the dysregulated genes may function as either effectors or promoters of ULMs growth, they are likely secondarily induced and indirectly responsible for tumor growth into morbid and symptomatic ULMs.

MicroRNAs are a class of small, non-coding regulatory RNAs. In cells, transcribed microRNA precursors (pri-microRNAs) undergo multistep biogenesis to form mature microRNAs of 18–25 nucleotides in length [3]. MicroRNAs regulate a high number of biological processes including cell proliferation, differentiation and cell death during development by sequence-specific targeting of particular mRNAs [4] and are aberrantly expressed in many solid tumors [5], [6], [7], [8], [9], [10] including uterine ULMs [11], [12], [13]. According to miRBase (Release 15), a total of 940 human microRNAs have been identified [14]. Cloning approaches and computational predictions have indicated that there are possibly even more microRNA encoding loci in the human genome [15]. Largely based on ectopic expression experiments and computational algorithms for prediction of microRNA target sites in mRNA sequences, it is estimated that each microRNA may regulate hundreds of genes at the post-transcriptional and translational levels [16].

Recently, studies revealed that a subset of microRNAs are significantly dysregulated in ULMs compared to matched myometria [11], [12], [13]. Many of these microRNAs are also associated with other neoplasms, indicative of their roles in tumorigenesis in general [13]. It is thus important to establish the roles played by the highly dysregulated microRNAs in ULMs pathogenesis through regulation of specific target genes with key roles in tumorigenesis. We and others demonstrated that the *let-7* microRNA family could functionally repress *HMGA2* expression [13], [17], [18], [19]. These findings prompted us to explore the broader relationship between other dysregulated microRNAs and their target genes exhibiting aberrant expression in ULMs.

In this study, we compared global microRNA expression patterns with the expression of their predicted target genes, at both mRNA and protein levels, in paired sets of ULMs and matched myometria, focusing primarily on the inverse association between the levels of microRNAs dysregulated in ULMs and the expression of their predicted target genes. We found that in uterine ULMs the levels of the most dysregulated microRNAs show an inverse association with the expression levels of many predicted target genes, and that they may affect multiple homeostatic pathways and functions. Next, by correlating comparative genomic hybridization (CGH) results and microRNAs data, we found that dysregulation of certain microRNAs with established roles in cancer could be due to the underlying genomic alterations. Finally, we show that selected predicted target genes can be validated as functional targets of specific microRNAs *in vitro*. In summary, our study offers a catalogue of microRNA, mRNA and protein expression alterations genetically or functionally related to the pathogenesis of human uterine ULMs.

## Materials and Methods

### Patients and Tissue Samples

This study included 55 uterine ULMs of usual type from 41 patients, as published elsewhere [13]. All clinical information is well documented. Among 55 ULMs, 36 ULMs and matched myometria were collected to prepare high density tissue microarray (TMA) from formalin-fixed paraffin embedded tissue (FFPE), and 24 ULMs and matched myometria were collected to prepare total RNA from snap frozen tissue, and used for RT-PCR validation. Among them, eight were selected for comparative genomic hybridization and five cases were selected for gene expression analysis (see below). The patient and tissue sample information is summarized in Table 1. The study was approved by the New York University and Northwestern University institutional review boards.

**Table 1:** Summary of patient and tissue sample information.

Case No.	Ethnic	Age (yrs)	Uterine weight (gm)	Tumor size (cm)	No. Tumors (n)	Profile			TMA	RT-PCR
						miRNA*	mRNA	CGH		
C4	Black	45	750	10.5	5	yes	yes	yes	yes	Yes
C7	Black	48	850	11	5	yes		yes	yes	yes
C18	Black	48	2500	12	30	yes	yes	yes	yes	yes
C19	Black	48	3800	24	8	yes	yes	yes	yes	yes
C21	Black	43	1000	9	11	yes				yes
C32	Black	35	1300	17	6	yes	yes	yes	yes	yes
C36	Black	42	1050	15	42	yes	yes	yes	yes	yes
C41	Black	51	900	9	50	yes		yes	yes	yes
C51	Black	39	2210	14	105	yes		yes	yes	yes
C52	Black	52	5400	26	20	yes			yes	yes
C56	Black	50	600	12	10	yes			yes	yes
C57	Black	50	1140	8	102	yes			yes	yes
C58	Black	50	440	6	52	yes				
C59	Black	45	950	10	20	yes				
C68	Black	50	840	8	10	yes				
C6	White	52	1200	17	3	yes			yes	yes
C9	White	38	450	12	2	yes			yes	yes
C10	White	52	1200	11	10	yes			yes**	yes
C13	White	44	475	11.5	3	yes			yes	yes
C15	White	38	2100	14	5	yes			yes	yes
C20	White	46	1200	11	2	yes			yes**	yes
C22	White	53	1875	14	5	yes			yes	yes
C26	White	46	650	8	25	yes			yes	yes
C29	White	56	1500	11	5	yes			yes	yes
C46	White	51	1300	11	51	yes			yes	yes
C47	White	47	1150	12	15	yes			yes	yes
C53	White	54	2400	20	3	yes				yes
C61	White	48	1200	10	10	yes			yes	
C66	White	45	850	11	3	yes			yes	
C67	White	54	650	8	3	yes				
C3	Asian	44	800	7	22	yes				
C24	Asian	42	890	13	2	yes			yes	
C27	Asian	56	950	10.5	5	yes			yes	
C24	Asian	40	1100	13	12	yes				
C40	Asian	41	950	8	25	yes			yes	
C42	Asian	51	900	12	5	yes			yes	
C1	Hisp	41	1100	11	10	yes			yes	
C5	Hisp	48	550	11	6	yes			yes	
C14	Hisp	48	1100	14	5	yes			yes	
C16	Hisp	50	2100	11	40	yes			yes	
C23	Hisp	47	450	8.5	2	yes				

*Wang et al. 2007.

**Two tumors (Large and small).

### Cell lines

Four uterine smooth muscle cell lines were used for the study. They include two immortalized uterine leiomyoma cell lines, ULM-3401 (obtained from an intramural leiomyoma of 45 year old African American woman), immortalized by the introduction of stable human telomere terminal transferase expression (*hTERT*) and UtLM, obtained from Dr. Dixon's lab (for details please see reference [20]). ULM-3401 exhibits a high level of HMGA2 expression. Two uterine leiomyosarcoma cell lines SK-LMS-1 and SK-UT-1 were obtained from ATCC.

### Gene expression analysis

The mRNA expression profiles of 5 ULMs and matched myometria were examined. Affymetrix® HG-U133A GeneChips were used to generate expression data for 22,000 probe sets identifying 18,400 transcripts of 14,500 genes. ULMs and matched myometrial tissues were homogenized in lysis buffer (Ambion, Austin, TX). Following the manufacturer's protocol, total RNA extraction and clean-up were performed using an Ambion RNA purification kit (Ambion, Austin, TX). RNA samples were processed following the Affymetrix protocol (Affymetrix Inc., Santa Clara, CA). In brief, 8 µg of total RNA was amplified, biotin labeled, and hybridized to the Affymetrix HG-U133A GeneChips (Affymetrix Inc.). The gene expression data from ULMs previously published by Hoffman et al [21] were obtained from the NCBI Gene Expression Omnibus database under the accession number GSE593, with sample IDs GSM9093 through GSM 9102. This data set was used as reference to compare with our gene profiling data. The raw array data generated in this study are deposited in NCBI GEO database under the identifier GSE 23112.

### Comparative genomic hybridization (CGH)

Eight ULMs and matched myometria from African American women were selected for array CGH analysis (Table 1). Genomic DNA from ULMs were used as the “test” DNA and matched myometria as the “reference” DNA during the hybridization reaction. Human Genome CGH 44K Microarrays (ID:014950) from Agilent Technologies were used according to the instructions of the array manufacturer. Briefly, the test and reference DNAs were digested with Alu I and Rsa I (Promega), and purified with the QIAprep Spin Miniprep kit (Qiagen). Test DNA (500 ng) and reference DNA (500 ng) l were labeled with either Cy3-dUTP or Cy5-dUTP (Perkin Elmer) using the Bioprime Array CGH Genomic Labeling kit (Invitrogen), and hybridized with 2× Hybridization buffer (Agilent, Palo Alto, CA), 10× blocking agent (Agilent,), and Human Cot-1 DNA (Invitrogen) in an Agilent SureHyb chamber for 24 hours at 65°C. After four washing steps, all slides were scanned by Agilent Scanner (G2565CA). Raw data were obtained by Agilent Feature extraction software 9.0, and then imported into Agilent CGH analytics 3.5 software for analysis.

DNA copy number changes were detected by CGH analytics software 3.5 (Agilent). The ADM-1 statistical algorithm was used with a sensitivity threshold of 6.0 and a moving average window of 1 Mb. In order to determine that there was a copy number change in a particular locus, three criteria had to be met. These were positive calls by the software, presence of 10 consecutive probes pointing in the same direction, and 1.5 fold average fold difference in the test DNA compared to the reference DNA.

### Expression data and bioinformatics analyses

GeneSpring GX11 (Agilent Technologies, Palo Alto, USA) and TM4 Microarray Software Suite [22] were used to identify differentially expressed mRNAs in ULMs. The raw array intensities (CEL files) were normalized using robust multichip average (RMA) and filtered using Significance Analysis of Microarrays (SAM) with a false discovery rate of 5%. The TargetScan and PicTar microRNA target prediction algorithms were then used to identify, on the mRNAs that passed the statistical filtering, putative targets of the 10 most highly dysregulated microRNAs (top five up and top five down). The genes identified by both prediction algorithms were further analyzed by function and pathway using the Database for Annotation, Visualization and Integrated Discovery (DAVID), available from http://david.abcc.ncifcrf.gov/ [23]. Unsupervised hierarchical clustering was performed to visualize the correlations between microRNAs and target mRNA/protein levels. For visualization of global microRNA::mRNA interactions in ULMs, box plots were used as a convenient way of graphically depicting the regulation of the mRNA targets of the 5 most highly upregulated and 5 most downregulated microRNA. The arms of the box plot represent the smallest and largest observation, the box is delimited by the lower and upper quartile, and the line through the box represents the median of the observations. Gene set enrichment analysis (GSEA) in ULMs versus matched myometria was performed according to the authors' guidelines published at the Broad Institute web pages (http://www.broadinstitute.org/gsea/index.jsp), using C2 (curated gene sets) and C5 (Gene Ontology gene sets) collections using Signal2Noise ratios.

### MicroRNA transfection

MicroRNA oligonucleotides were used at a concentration of 60 pmol/well for a 6-well plate. To estimate transfection efficiency, the negative control Block-iT (Fluorescent double-stranded random 22mer RNA from Invitrogen (Carlsbad, CA)) and positive control TSC2 siRNA (Invitrogen, CA) were used. Mature microRNAs mimics and inhibitors from *let-7c* and *miR-296* were purchased from Dharmacon Inc. (Lafayette, CO). Cells receiving only the tagged random sequence (Block-iT) were used as non-specific references at all data points. Following transfection, cells were harvested and analyzed at the indicated times.

### shRNA miR-200a infection

Human *miR-200a* shRNA in pGIPZ was prepared. Lentivirus expressing *miR-200a* shRNA were produced in HEK293T cells packaged by pMD2G and psPAX2. For stable infection, 4×10^4^ cells/mL of UtLM cells were plated in each well of 6-well plates in 2 ml medium without antibiotics. After overnight incubation, media was replaced with 1 ml Opti-MEM® I Reduced-Serum Medium containing 12 µg/mL polybrene per well. 50 µL of concentrated lentiviral particles were added to each well. After 48 hours infection, fresh media was added containing 2 µg/ml puromycin. Fresh media with puromycin was replaced every 3–4 days. Single clones were picked after two weeks of puromycin selection. Stable miR-200a expression was validated by RT-PCR (see below).

### Cellular proliferation assay

UtLM cells were seeded in 24-well plates in triplicate wells at densities of 1×10^4^ per well. Cell proliferation was monitored at 24, 48, 72 and 96 hrs using the colorimetric MTS assay (CellTiter 96® Aqueous Assay, Promega).

### Luciferase transfection assays

Cell lines were transfected with 200 ng luciferase reporter PGL-3 control (Promega, Madison, WI), or pGL-3 HMGA2-3′UTR construct and 1ng of the pRLuc internal control plasmid (Biosignal, Montreal, QB). The luciferase expression was determined as recommended by Promega (Madison, WI).

### Western blot analysis

The culture cell samples were homogenized at 4°C in a protein lysis buffer. Equal amounts of total protein from each sample were resolved through a 10% SDS–PAGE gel and then transferred to a PVDF membrane (Perkin Elmer Life Scientific Inc.). Development of the immunoblot with antisera against TSC2 (from Dr. Mizuguchi [24], [25]) was tested and a single specific HMGA2 band at 25 kDa was detected, as previously described.

### RT-PCR

For detection of mature microRNAs, *mirVana* qRT-PCR Primers and the *mirVana* qRT-PCR Detection Kit (Ambion, Austin, TX) were used and optimized according to the abundance of microRNAs in the tested tissue samples. Primers for 11 *let-7* and 5 *miR-200* predicted target genes are summarized in Table S1. The abundance of cDNA products was normalized to the internal controls of small nuclear RNA *U6* and *α-Actin*.

### Tissue Microarrays, immunohistochemistry and scoring

All tissue sections were reviewed and cellular areas of tumor were selected for TMA analysis. Six 0.6 mm tissue cores were collected from each case, including three cores of ULMs and three cores of matched myometria. The technical details and reliability of the data have been described previously [26]. In brief, 432 tissue cores from 36 ULM with matched myometria were arrayed into one recipient paraffin block. The tissue cores were arrayed in a random distribution of cases.

Antibodies selected for this study included sixteen protein markers and are listed in Table S2. The detailed protocols and conditions used for each antibody followed the manufacturers' recommendations which were described previously [27]. In brief, the paraffin-embedded tissue array blocks were sectioned at 4 µm. Antigen retrieval was performed by either heat-induced epitope retrieval or by proteolytic enzyme digestion as previously described. All immunohistochemical staining procedures were performed on a Ventana Nexus automated system (Tucson, Arizona, USA).

Stained TMA slides were graded jointly by two pathologists using a visual semiquantification method (optical density of the immunoreactivity) depending on the staining characteristics of the antibody. The net gain or loss of immunoscores was calculated for a specific immunomarker from each individual ULM in comparison with matched myometrium (net value = tumor immunoscore − myometrial immunoscore). Each net value gave positive or negative scores.

## Results

Largely based on ectopic expression studies and computational analyses, it has been predicted that microRNAs can regulate at least 30% of gene transcripts. Several recent studies have compared microRNA expression with the levels of corresponding predicted target genes in normal and tumor tissues at a global level. Our previous findings of microRNA dysregulation in ULMs led us to further examine whether these dysregulated microRNAs correlated with abnormal expression of their predicted target genes at both transcriptional and translational levels.

### Genome-wide correlation of dysregulated microRNAs and their predicted mRNA targets

We had reported that a subset of 45 microRNAs were differentially expressed between ULMs and matched myometria [13]. To test whether altered expression of microRNAs in ULMs are responsible for dysregulation of the predicted target gene expression, we selected five ULMs and matched myometria (for which we had identified microRNA profiles, see Table 1) for gene expression analysis using Affymetrix U133A arrays. The five cases selected for the gene expression study were marked by the most significant microRNA dysregulation in ULMs and the highest magnitude of their modulation (Figure S1). In particular, we selected ULMs of large size (>10 cm) and from African American women. As previous stated by Aslan et al [2], the gene expression profiles in ULMs varied widely among studies, affected by the clinical setting, methods and platforms. We selected to also analyze the NCBI GEO GSE593 ULM profiling along with the data from this study due to the same sample size, patient age and the microarray platform used [21].

Applying significance analysis of microarray to the combined set of our 5 pairs of ULM and matched myometria and the GSE593 data, we identified 2674 probe sets (1,117 up- and 1,557 down-modulated) that were significantly different between ULMs and matched myometrium controls (Figure S1).

We used a combination of two microRNA prediction methods, TargetScan and PicTar, to search for all predicted gene targets of the 5 most highly upregulated (*let-7s*, *miR-21*, *miR-23b*, *miR-27a and miR-30a*) and downregulated (*miR-29b*, *miR-32*, *miR-144*, *miR-197 and miR-212*) microRNAs [13]. Both mRNA profiling data sets (ours and GSE593) were used for the microRNA target prediction.

**Figure 1:**
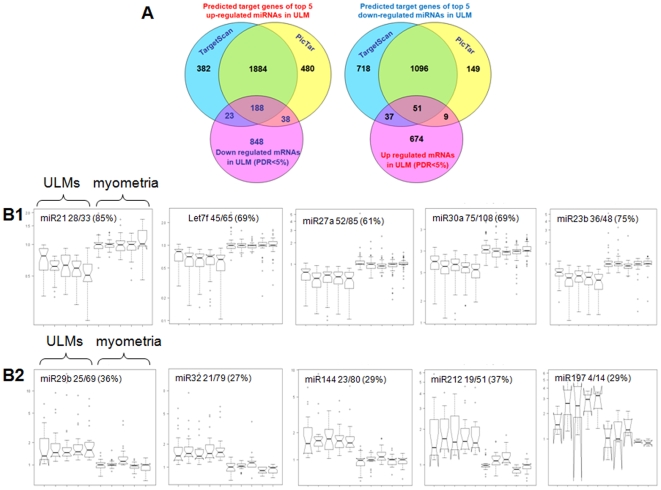
Predicted target genes or microRNAs in uterine ULMs. **A** Predicted target genes for the 5 most highly upregulated (left) and 5 most highly downregulated microRNAs (right). TargetScan (light blue) and PicTar (yellow) identified 1884 genes as predicted targets of the 5 most highly upregulated microRNAs (*let-7s*, *miR-21*, *miR-23b*, *miR-27a and miR-30a*). Among 1079 significantly downregulated genes in ULMs (pink circle), 188 (intersection of the three circles) are the best predicted targets of these upregulated microRNAs. **B** Differential expression of the predicted target genes for each of the 5 most highly upregulated microRNAs (B1) and downregulated microRNAs (B2) in ULMs and matched myometria. Each box plot represents the average level of predicted target gene expression. Expression of microRNA targets is plotted as RMA-normalized, median centered and log2-transformed, relative abundance levels (Y-axis), with a baseline = 1 corresponding to median centered normal myometrial samples.

There were a total of 2995 and 2060 TargetScan and PicTar-predicted genes of the top 5 upregulated and 5 top downregulated microRNAs, respectively (Figure 1A). Utilizing the expression data from these five cases, we identified 2674 mRNAs that were significantly dysregulated in ULMs (SAM, FDR<5%, Figure 1A). Among these significantly dysregulated mRNAs, 249 downregulated mRNAs were predicted targets of the 5 upregulated microRNAs and 97 upregulated mRNAs were predicted targets of the 5 downregulated microRNAs (Figures 1B). Together, they represented 13% of 2674 genes found to be significantly dysregulated in ULMs. The Box Plot analysis and significance analysis revealed a trend of overall inverse association between the predicted target genes and either up- or down- regulated microRNAs (Figure 1B). Upregulated predicted gene targets of downregulated microRNAs were significantly enriched in comparison to the overall pool of significantly dysregulated targets (0.05>p>0.01). The findings suggest that the most up and down regulated microRNAs in ULMs may regulate the expression of their predicted target genes at the level of mRNA stability as previously reported [28].

At the level of individual matches between microRNAs and the inversely modulated, predicted targets, a rather small proportion of the target genes were inversely correlated with a considerable correlation coefficient (ranging from r = −0.5 to −0.95, see Table 2). Small sample size and important biological variables such as microRNA subcellular compartmentalization and the fact that levels of mature microRNAs do not necessarily reflect their functional impact may be the reason for the decreased rates of significant negative correlation of individual microRNA and mRNA expression patterns.

**Table 2:** Correlation analyses of top 10 most highly dysregulated miRNAs and their predicted target genes in 5 large ULM of black women based on global gene expression profiles.

miRNA	Symbol	Target gene Name	AFFY ID	Correlation coefficient (n = 5)
*let7c*	TRIB1	tribbles homolog 1 (Drosophila)	202241_at	−0.437
*let7f-2*	PLCB4	phospholipase C, beta 4	203896_s_at	−0.648
*miR-21*	BRD1	bromodomain containing 1	215460_x_at	−0.878
	SKI	v-ski sarcoma viral oncogene homolog (avian)	204270_at	−0.435
*miR-27a*	ANK2	ankyrin 2, neuronal	202921_s_at	−0.942
	RAB11FIP	RAB11 family interacting protein 1 (class I)	219681_s_at	−0.869
	GATA2	GATA binding protein 2	209710_at	−0.890
	FOSB	FBJ murine osteosarcoma viral oncogene homolog B	202768_at	−0.662
	PPARG	peroxisome proliferative activated receptor, gamma	208510_s_at	−0.692
*miR-30a-5p*	SMARCD2	SWI/SNF related, matrix associated, actin dependent regulator of chromatin, subfamily d, member 2	201827_at	−0.760
	SLC29A3	solute carrier family 29 (nucleoside transporters), member 3	219344_at	−0.604
	HLF	hepatic leukemia factor	204755_x_at	−0.817
	MAP3K5	mitogen-activated protein kinase kinase kinase 5	203836_s_at	−0.881
	TNXA	tenascin XA pseudogene	216339_s_at	−0.598
*miR-23b*	PPARG	peroxisome proliferative activated receptor, gamma	208510_s_at	−0.766
	HIVEP2	human immunodeficiency virus type I enhancer binding protein 2	212641_at	−0.828
	GATA2	GATA binding protein 2	209710_at	−0.863
	FOSB	FBJ murine osteosarcoma viral oncogene homolog B	202768_at	−0.641
*miR-29b*	BMP1	bone morphogenetic protein 1	202701_at	−0.942
	RARB	retinoic acid receptor, beta	205080_at	−0.625
	FAM131B	family with sequence similarity 131, member B	205368_at	−0.814
	NASP	nuclear autoantigenic sperm protein (histone-binding)	201969_at	−0.940
	TGFB3	transforming growth factor, beta 3	209747_at	−0.644
*miR-32*	NFIB	nuclear factor I/B	211467_s_at	−0.785
	FLI1	Friend leukemia virus integration 1	210786_s_at	−0.805
	FHL3	four and a half LIM domains 3	218818_at	−0.799
*miR-212*	PRKD1	protein kinase D1	205880_at	−0.308
*miR-144*	PTHLH	parathyroid hormone-like hormone ; parathyroid hormone-like hormone	211756_at	−0.239
*miR-197*	RNPC1	RNA-binding region (RNP1, RRM) containing 1	212430_at	−0.803
	TNRC5	trinucleotide repeat containing 5	217931_at	−0.566

### Correlation of microRNA expression with protein expression in pair-matched tissue samples of ULMs and myometria

To further investigate the correlation of microRNAs and predicted targets in ULMs, we examined the predicted target gene products by immunohistochemistry analysis to establish the effects of microRNAs on the synthesis of target proteins. We utilized ULMs and matched myometria from 36 cases, from which global microRNA expression profiles had been obtained [13] (Table 1). A total of 16 proteins (Table S2) that were known to be significantly dysregulated in ULMs [27], [29] were selected for immunohistochemical analysis. The relative expression level of each protein was scored in a semiquantitative manner in ULMs and in matched myometrial controls (see Methods). The net changes of the selected gene products (Figure S3) were analogous to our previously published studies in ULMs [27], [29].

**Figure 2:**
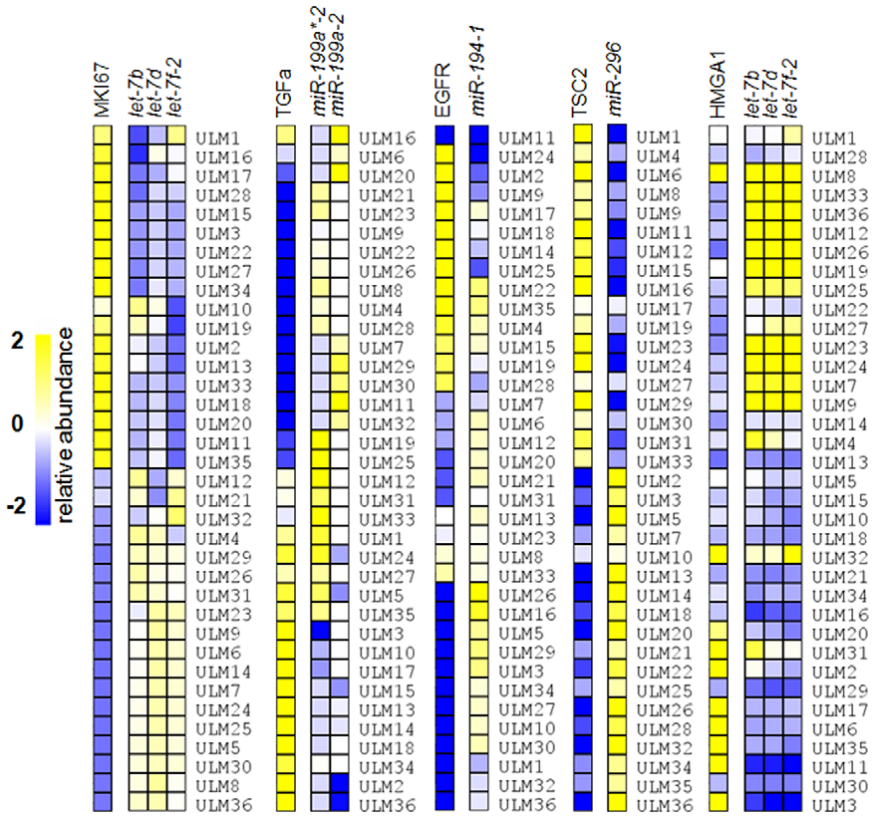
Correlation of the selected microRNAs and their predicted target gene products (proteins) in 36 ULMs. The negative correlation of microRNA and the target proteins are illustrated by inverse correlation of regulating miRNAs and their select targets. The gene expression levels are depicted by the intensity of yellow (overexpression), white (no change) and blue (underexpression) color. In each panel, correlated microRNAs and the target proteins are shown on the top and tumor IDs are on the right.

The Ki-67 index in ULMs in comparison to the matched myometria can be used to evaluate tumor growth rate. Correlation of *let-7* microRNA levels in 36 ULMs established an inverse association with the Ki-67 index (MKI67 protein expression) (Figure 2).

Moderate individual negative correlations with microRNAs were found for EGFR and *miR-194-1*, TGFα and *miR-199a-2* (r = −0.39 and r = −0.58, respectively), however, the overall pattern appeared entirely inversely correlated (Figure 2). Most other gene products, the patterns of which correlated with their targeting microRNAs, exhibited similar negative correlation (r = −0.2 to −0.4) (data not shown). These findings validate the targets and prediction-based correlative analysis of inverse associations of microRNA and tumor-associated protein expression levels.

### Genomic alteration and microRNA expression

To evaluate whether selected dysregulated microRNAs in ULMs associated with specific genomic alterations, we performed comparative genomic hybridization (CGH). We selected 8 cases of ULMs for the study (Table 1). By comparison to matched myometria, few genomic alterations were found (Table 3, Figure S2). In general, the loss of genomic material was the only finding in all eight cases. Chromosomal regions of 1p36, 3p11, 3q22 and 6p were the most commonly altered, with 1p36.33-p36.23 and 3q26.1-q27.2 established as regions of deletion overlap between ULMs derived from patients B4, B9 and B1, B4, respectively. Interestingly, members of cancer-inhibitory miRNA family *miR-200a*, *miR-200b*, *miR-429* and *miR-551a* are located in the region of loss at 1p36 while the 3q26-27 region harbors *miR-15b*, *miR-16-2* and other microRNAs including *miR-1263*, *miR-720*, *miR-551b*, *miR-569*, *miR-1224*. We also found that additional members of the oncogenic miR15/16 family (miR-15a and miR-16) as well as the members of the miR-17-92 polycistron were lost with the 13q12.12-q33.2 region of a single patient B4 (Table 3).

**Table 3:** Comparative genomic hybridization analysis and associated microRNAs in eight large leiomyomas of black women.

Patient	Chr. bands	Size (Mb)	Aberrations	MiRNAs in regions of loss*
**B1**	3p11.2-p11.1	0.55	loss	*-*
	3q13.31-q21.2	9.90	loss	*miR-198*
	3q22.2-q27.2	49.60	loss	*miR-15b, miR-16-2, miR-1263, miR-720, miR-551b, miR-569, miR-1224*
**B2**	No change	-	-	*-*
**B4**	1p36.33-p34.3	38.66	loss	***miR-200b, miR-200a*** *, miR-429, miR-551a, miR-34a, miR-1290, miR-1256, miR- 552*
	3q26.1-q29	37.68	loss	*miR-15b, * ***miR-16-2*** *, miR-1263,miR- 720, miR-551b,miR- 569, miR-1224, miR-1248, miR-28, miR-944, miR-570, miR-922*
	6q13-q24.3	76.21	loss	*miR-30c-2, miR-30a, miR-2113, miR-587, miR-548b, miR-588, miR-548a-2*
	13q12.12-q33.2	82.49	loss	*miR-320d-1, miR-621, miR-16-1, * ***miR-15a*** *, miR-1297, miR-622, * ***miR-17*** *, miR-18a, * ***miR-19a, miR-20a*** *, miR-19b, * ***miR-92a-1*** *, miR-623*
**B5**	No change	-	-	*-*
**B6**	No change	-	-	*-*
**B7**	22q11.1-q13.33	35.09	loss	*miR-648, * ***miR-185*** *, miR-1306, miR-1286, miR-649, * ***miR-301b*** *, * ***miR-130b*** *, miR-650, miR-548j, miR-658, miR-659, miR-1281, miR-33a, miR-1249, * ***let-7a-3, let-7b***
**B8**	No change	-	-	*-*
**B9**	1p36.33-p36.23	7.09	loss	***miR-200b*** *, miR-200a, miR-429, miR-551a*
	3p12.3-p11.1	10.24	loss	*-*
	6p25.3-p22.2	24.79	loss	*miR-548a-1*
	6p12.3-p11.1	11.61	loss	***miR-206*** *, miR-133b*

*: **bold** = miRNA detected as downregulated in the patient.

### Candidate role of the loss of miR-200 family and miR-15 and miR16 in ULMs

Loss of *miR-200* family is associated with epithelial and mesenchymal transition (EMT) and aggressive tumor phenotypes of ovarian cancer [30], [31]. We observed that *miR-200a* and *miR-200b* were significantly down regulated in 51 ULMs (net loss of −0.36±0.11 and −0.45±0.06, respectively). To further explore the potential significance of the deletion overlap involving the miR-200 family, we examined gene expression in patients and found that a number of TargetScan-predicted targets of either *miR-200a* or *miR-200b* were collectively upregulated in the tumor compared to unaffected pair-matched myometrium (∼180 genes ranging from 1.25–10.3 fold upregulation). Among these were genes with established roles in cancer and cell death regulation: MAF (v-maf musculoaponeurotic fibrosarcoma oncogene), CTBP2 (C-terminal binding protein 2), antiapoptotic BCL2 (B-cell CLL/lymphoma 2), CITED2 (Cbp/p300-interacting transactivator), LASS6 (LAG1 homolog, ceramide synthase 6), PHF21A (PHD finger protein 21A), TSC22D1 (TSC22 domain family, member 1), ATXN1 (ataxin 1), JUN (jun oncogene) and NFIB (nuclear factor I/B). Focused pathway analysis (using GO, KEGG, Biocarta and Panther databases) of the predicted *miR-200* family targets that are consistently upmodulated in ULMs including patient B4 (Table S3) implicates categories of regulation of transcription proliferation and cell cycle control, actin cytoskeleton and adherens, tight, gap and focal adhesion junction remodeling, as well as cancer related signaling pathways (MAPK, RAS, WNT, NOTCH, TGF-β, VEGF).

Similarly, loss of *miR-15/miR-16* cluster is associated with aggressive tumor growth [32]. The findings indicated alteration of these two genomic regions may be related to the tumorigenesis of ULMs. We have evaluated the expression levels of TargetScan predicted mRNA targets and found that important transcriptional, signaling and other regulators of cell growth and survival linked to cancer were also collectively upmodulated in patient B4, among them FOXO1A (forkhead box O1A (rhabdomyosarcoma)), BCL2 (B-cell CLL/lymphoma 2), TGFBR3 (transforming growth factor, beta receptor III (betaglycan, 300kDa)), MAP3K4 (mitogen-activated protein kinase kinase kinase 4), VEGF (vascular endothelial growth factor), TCF3 (transcription factor 3), EIF4E (eukaryotic translation initiation factor 4E), JARID2 (Jumonji, AT rich interactive domain 2), EVI5 (ecotropic viral integration site 5), IGF1 (insulin-like growth factor 1), WNT5B (wingless-type MMTV integration site family, member 5B). Detailed pathway analysis of the upregulated targets of the lost *miR-15/16* family identified biological categories of pathways in cancer, endometrial cancer, transcription, melanoma, apoptosis, signaling pathways including insulin, MAPK, mTOR, VEGF, ErbB, JAK/STAT signaling, and cell-cell adhesion and cytoskeleton remodeling (see Supplemental Table 3)

We also identified a subset of upregulated genes that are predicted as targets of both *miR-200a/b* and *miR-15/16* microRNAs from the regions of deletion overlap on Chr1 and Chr3 (Table 3), including ACTR1A, BACH2, BCL2, CDC14B, CLASP1, CYP26B1, E2F3, EVI5, FUBP1, IKBKB, IRS2, IRS2, LRIG1, OTUD4, PCDH9, PCDH9, PELI2, PHF21A, PPAP2B, SLC2A3, SNTB2, TMCC1 and TUBB.

These findings indicated that alteration (loss) of two overlapping genomic regions (7.09 Mb of Chr1 1p36.33-p36.23 and 24.56 Mb of Chr3 3q26.1-q27.2 harboring cancer related miRNAs may be related to the tumorigenesis of a subset of ULMs via deregulation of the *miR200a/b* and *miR-15/16* gene targets and in part this process may be due to the loss of convergent inhibitory action of the *miR-200 family* and *miR-15* and *miR-16* on a small group of the same downstream target genes.

### Functional correlation of microRNAs and mRNA expression in cultured leiomyoma cell lin

**Figure 3:**
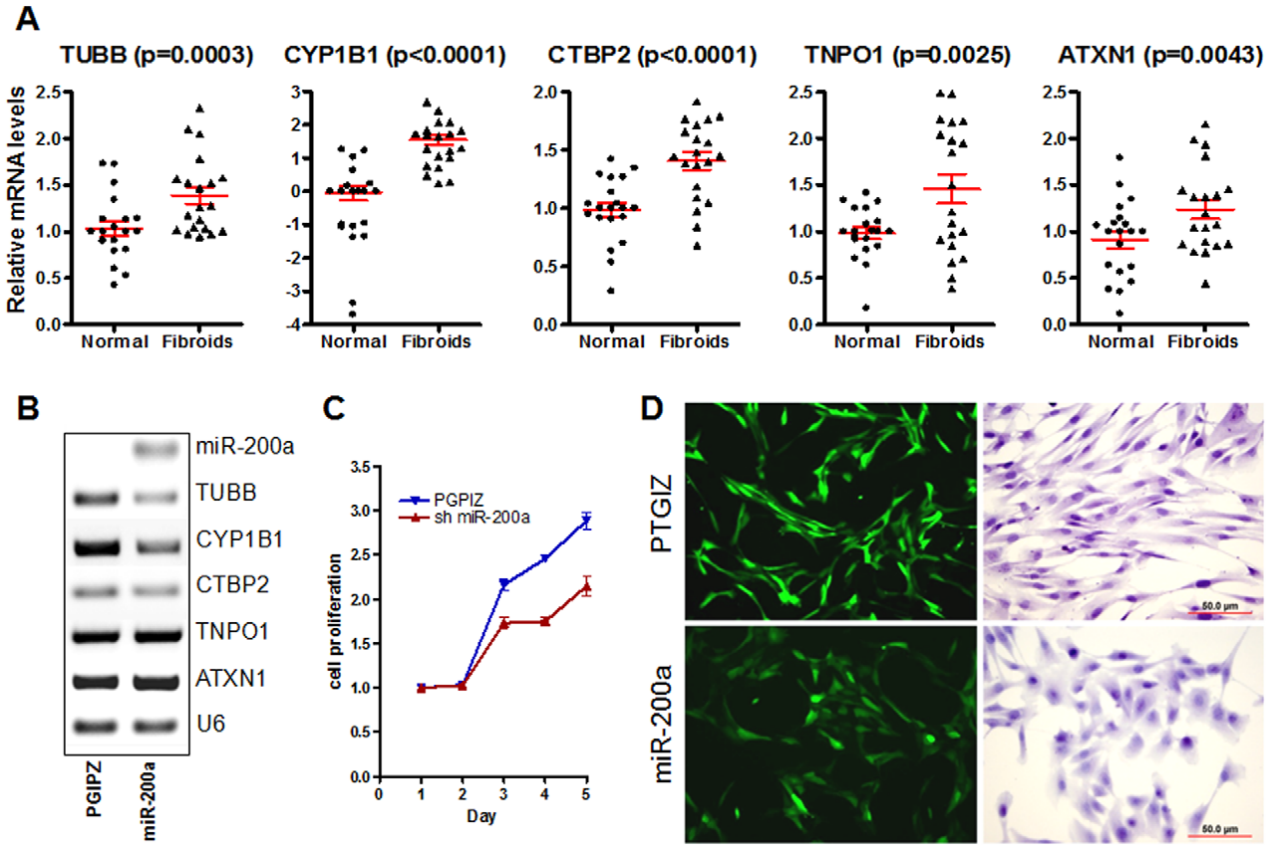
*MiR-200* predicted target gene analysis in uterine ULMs. **A** Scatter plot analysis of relative mRNA expression in five *miR-200* predicted target genes in 10 ULMs and matched myometria (our data and GSE593). Red bars indicate the mean and standard error of measurement. **B** RT-PCR analysis of expression of five *miR-200* predicted target genes in ULMs cell line UtLM with stable miR-200a expression (see Methods) and control (vector PGIPZ only). Repression of TUBB, CYP1B1 and CTBP2 can be readily appreciated. **C** Growth curves illustrate significantly reduced proliferative rate in UtLM cell line with *miR-200a* overexpression in comparison UtLM cell line with vector control only (PGPIZ). D. Photomicrographs illustrate stable viral control (upper panels) and *miR-200a* (lower panels) expression in UtLM cell lines. The stromal to epithelial morphology transition in UtLM cell line with *miR-200a* overexpression is evident (lower panels).

As shown above, downregulation of *miR-200* family members appears a candidate event in leiomyomas in select patients due to loss of corresponding genomic DNA loci and broader reduction of *miR-200* expression in ULMs (Table 3 and Figure S2). We selected 5 target genes of *miR-200a* that were all significantly upregulated in ULMs (Figure 3A) for functional testing as direct targets and for their roles in cell phenotype changes. In the UtLM cell line with stable *miR-200a* overexpression, 3 of 5 mRNAs (TUBB, CYP1B1 and CTBP2 were suppressed by *miR-200a in vitro* (Figure 3B). Importantly and as predicted by pathway analysis (Table S3), overexpression of miR-200a in UtLM cells led to growth inhibition compared to mock infected controls (Figure 3C), and reverted the fibroblastoid morphology towards more pronounced epithelial phenotype (Figure 3D), consistent with the established role of *miR-200* family in epithelial-mesenchymal transition [33]. Collectively, these findings suggest that the loss of *miR-200* family, identified by miRNA profiling and a CGH analysis, together with upregulation of its target genes may contribute to the tumor growth and underlie the mesenchymal character of ULMs.

**Figure 4:**
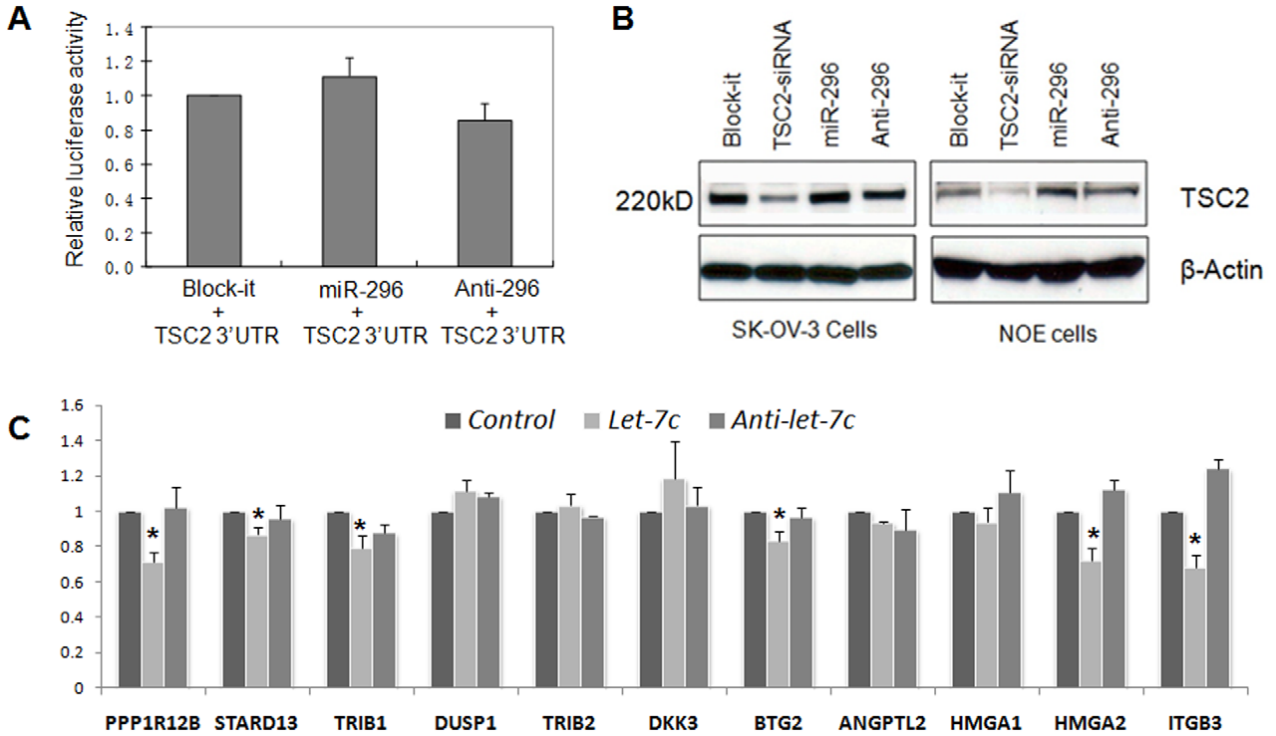
Analysis of the *miR-296* predicted target gene *TSC2* and 11 *let-7* predicted target genes *in vitro*. **A** Transient transfection analysis for luciferase reporter expression with *TSC2* 3′UTR in the presence and absence of *miR-296*. **B** Immunoblotting analysis of transient transfection analysis of miR-296 for TSC2 expression. *TSC2* siRNA was used as a positive control antagonizing *TSC2* expression. Block-iT = nonfucntional small RNA control. β-Actin was used as protein loading control. **C** Relative expression of *let-7* predicted target genes (listed above) (y-axis) in transient transfection of nonfunctional small RNA (Block-iT (Controls), *let-7c* mimic and *let-7* inhibitor (*Anti-let-7*). The relative expression levels were obtained in three cell lines of immortalized ULM cell line (ULM-3401), leiomyosarcoma cell lines (LMS-1, UT-1) (see Methods). T-bars indicate standard error of measurement. * = p value<0.05.

We previously found that the product of *TSC2* gene (tuberin) was significantly down regulated in ULMs [27], [34]. Downregulation of *TSC2* was also found in this study (Figure S3). By correlation analysis, as illustrated in Figure 2, we found that predicted regulatory *miR-296* was inversely correlated with TSC2 protein in 36 ULMs. *TSC2* contains 41 exons with a very short 3′ untranslated region (3′UTR, <110 nt). The short *TSC2* 3′UTR may prevent microRNA regulation. However, in the *TSC2* 3′UTR immediately adjacent to the stop codon, there is a highly conserved sequence that harbors the complementary sites of *miR-296* and a few other microRNAs. To study whether *TSC2* is the target of *miR-296*, we prepared a *TSC2* 3′UTR reporter construct and examined the luciferase activity by treated cells with control, *miR-296* mimic and inhibitor. There was no reduction of luciferase expression in cell treated with *miR-296* (Figure 4A). We further examined whether *miR-296* could inhibit TSC2 protein production. In comparison to *TSC2* siRNA, no significant protein reduction was noted in cells treated with *miR-296* (Figure 4B). The findings indicated that *TSC2* was not a direct target of *miR-296*. The inverse correlation of *TSC* and *miR-296* levels may thus be related to indirect or unrelated molecular mechanisms participating in ULM tumorigenesis.

We had reported that most members of the *let-7* family were dysregulated in ULMs [13]. To test whether *let-7* microRNAs could regulate other predicted target genes in addition to *HMGA2* in ULMs, we examined the expression of several predicted *let-7* target genes in the ectopically induced presence or absence of *let-7* microRNAs in uterine ULMs and leiomyosarcoma cell lines *in vitro*. TargetScan and PicTar databases [35], [36] predicted 65 target genes of *let-7* mRNAs that are significantly dysregulated in ULMs (Figure 1B1), of which 45 (69%) were downregulated. Of those, we selected 11 candidate targets involved in cell proliferation and extracellular matrix regulation that might be functionally associated with the pathogenesis of ULMs. By transient transfection and RT-PCR analysis (Table S1), we found that 6 of them can be repressed by *let-7s* (Figure 4C). Thus our findings indicate that a subset of the predicted target genes can be significantly repressed in experimental conditions. Importantly, this set of experiments indicates that follow-up biochemical functional studies are warranted to validate the prediction- and inverse correlation-based analyses of individual microRNA::mRNA relationships.

### Interpretation of focused miRNA::mRNA relationship compared to genome wide pathway analysis

**Figure 5:**
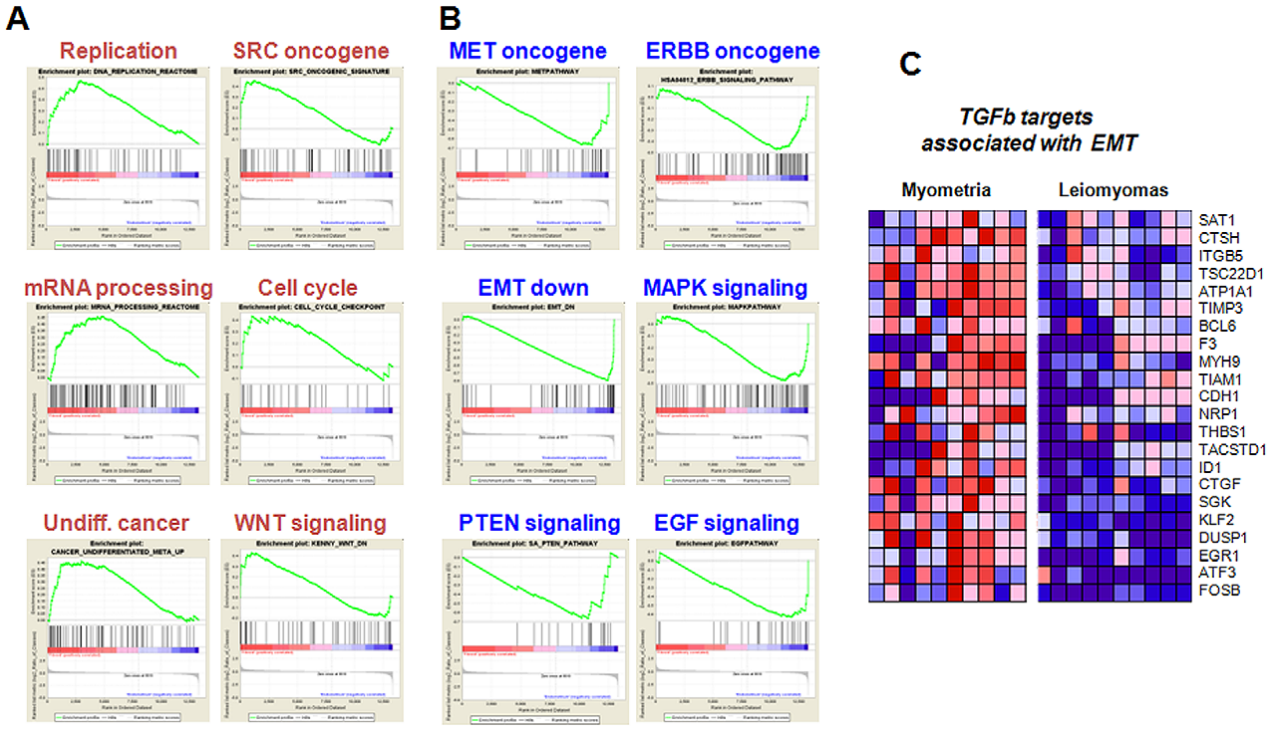
Gene Set Enrichment Analysis (GSEA) of mRNA profiling results from primary ULMs and matched myometria. The histograms show the distribution of select top GSEA molecular signatures from predefined C2 and C5 categories (accessible from the MolSig database at http://www.broadinstitute.org/gsea/msigdb/index.jsp). The leading edge (most significant genes) are shown as vertical bars accumulated either left and below the peak of green enrichment score plot (**A**) or right of the valley of the green plot (**B**), indicating the respective up- or down-regulated genes of each shown GSEA characterized by the highest enrichment score. **C** The leading edge (genes with the highest enrichment score) of the EMT-down category (shown in B) is shown as individual gene expression-based heat map and indicates downregulation of the TGF-β signal and of its canonical targets in ULMs.

We asked whether inverse correlation of the top 5 up- and down-regulated miRNA profiles with their target genes is sufficiently representative to identify top categories in comparison to broad data mining of the ULM mRNA profiles. To that end, we performed gene set enrichment analysis (GSEA, Figure 5) using the combined expression data from our and the GSE593 data and compared the results of top categories with the DAVID analysis of pooled 346 top miRNA targets (Fig 1B) presented in Table 4. A number of themes showed overlap, such as cell cycle and DNA replication control, proliferation control indicated by pathways such as MAPK, JAK/STAT, developmental signaling (WNT, TGF-β), and modulation of cellular processes associated with morphological and migratory properties of the tumors (cell-cell junction, cell adhesion and cytoskeleton remodeling) found in miRNA target analysis are complemented by MET, WNT and TGF-β signaling, major regulators of invasion and motility, found in both types of analysis. We thus propose that the reduced information contents resulting from functional interpretation of the relationships between a limited number of top regulated miRNAs and their inversely correlated mRNA targets sufficiently identifies key functional themes, with results comparable to the global, genome-wide mRNA analysis.

**Table 4:** KEGG/Biocarta analysis of functions of predicted target genes of miRNAs in ULM.

PATHWAY	miRNAs:	Up	Down	Gene Symbols
	mRNAs:	Down	Up	
MAPK SIGNALING ACVR1B, MEF2C		9		RPS6KA3, DUSP1, PDGFRB, RRAS2, MAPK14, MAP3K5,
FOCAL ADHESION GAP JUNCTION		7		MET, CCND2, TNXB, PDGFRB, RRAS2, ITGB3, CVR1B
REGULATION OF ACTIN CYTOSKELETON		6		ADCY9, ADCY3, CSNK1A1, GJA1, PDGFRB, RRAS2,
CYTOKINE-CYTOKINE		6		RDX, MYH9, PDGFRB, RRAS2, ARHGEF6, ITGB3,
RECEPTOR INTERACTION		5		MET, CSF1, KITLG, PDGFRB, ACVR1B,
ECM-RECEPTOR INTERACTION		5		TNXB, ITGB3, DTPRECK, TIMP3,
CELL CYCLE		5		CCND2, CCNH, RB1, ABL1, KITLG,
CALCIUM SIGNALING		4		ADCY9, ADCY3, PDGFRB, EDNRB,
JAK-STAT SIGNALING		4		SPRY1, STAT3, CCND2, STAT5B,
NFAT AND HYPERTROPHY OF THE HEART		4		CSNK1A1, MAPK14, HBEGF, MEF2C,
PPARá SIGNALING		3		RB1, DUSP1,STAT5B,
NFκB ACTIVATION		3		NR3C1,DUSP1,MAPK14,
TGF-â SIGNALING		3		CHRD, ACVR1B, SMAD7,
ADHERENS JUNCTION		2		MET, ACVR1B,
TIGHT JUNCTION		2		MYH9, RRAS2,
WNT SIGNALING		2		CCND2, CSNK1A1,
CELL ADHESION			3	NCAM1, CDH2, IGSF4,
INSULIN SIGNALING			3	SOCS2, PPARGC1A, FOXO3A,
CYTOKINE-CYTOKINE RECEPTOR INTERACTION			2	CXCL12, PDGFC,
FOCAL ADHESION			2	COL2A1, PDGFC,

## Discussion

Although it has been shown that individual microRNAs may regulate multiple genes, the current knowledge for microRNA::mRNA regulation has been largely obtained from the studies of the disruption of microRNA::mRNA interactions on a single gene basis. Recently, several attempts to examine microRNA::mRNA interactions globally have been pursued [37], [38], [39]. In our previous study of *TGF-β*-directed epithelial to mesenchymal transition, we explored a global approach to study the dynamics of microRNA::mRNA interactions [40]. The application of this genome-wide analysis of microRNA::mRNA expression in ULMs allows us to gain insight into the role of microRNAs in the regulation specific mRNAs and their protein products. In a correlation analysis between dysregulated mRNAs and the most highly of dysregulated microRNAs in ULMs, an overall negative correlation was established (Table 2 and Table S1, Figure 1).

Among many dysregulated microRNAs and target genes in leiomyomas, correlation of *HMGA2* and the *let-7* family has been well characterized [13], [17], [18], [19] and shown as biologically significant for leiomyoma growth [13], [41]. We expected to see broader dysregulation of additional genes in uterine leiomyomas due to regulation by specific microRNAs. In the selected predicted targets which we examined at the mRNA and protein levels, we found that some, but not all of them, appear to be regulated by microRNAs (Figures 2, 3, and 4). Our findings further support the notion that the selection of the predicted microRNA targets relies on the rank (strength) of the target prediction scores (Table S1) and that the functional regulation relies on the level of microRNA expression in the cell and convergent, synergistic effects of multiple microRNAs on one target gene. Varying functional validation outcomes of multiple genes tested as miRNA targets (Figures 3B and 4) importantly demonstrate the complexity of target gene regulation by microRNAs and indicate the need for detailed validation in prediction- and inverse correlation-based studies.

Uterine ULMs are the most common benign neoplasms, exhibiting complex patterns of gene dysregulation [1], [2]. The dysregulated genes are involved in many cellular and molecular functions, such as defects in angiogenesis [42], nuclear receptors and local growth factors [2], dysfunction of extracellular matrix [43] and TGF-β signaling [44]. The molecular mechanisms leading to the dysregulation of these genes has been largely unknown. As illustrated in Table 4, several tumorigenic pathways seem to be regulated by the most highly dysregulated microRNAs in ULMs. For example, at least nine predicted target genes of upregulated microRNAs in the Mitogen-activated protein kinase (MAPK) signaling pathway were downregulated. This finding was validated by the GSEA analysis of mRNA expression data (Figure 5B). MAPK signaling has been shown to play a significant role in tumorigenesis by controlling tumor growth, proliferation, differentiation, migration and apoptosis [45]. Activation of MAPK signaling is very common in many malignant neoplasms, allowing tumors to gain the capability of independent growth, insensitivity to anti-growth signals, unlimited replicative potential and the ability to invade and metastasize [45]. ULMs are relatively slow growing smooth muscle tumors and rarely, if ever, progress to malignancy. Repression of MAPK pathway genes by microRNAs in ULMs may represent a protective mechanism from fast growth and potential tumor progression.

Downregulation of many target genes of upregulated microRNAs including genes involved in focal adhesion (seven), gap junction (six), actin cytoskeleton (six) and extracellular matrix formation (five) have been identified (Table 4). These categories are related to cell remodeling, migration, growth and tumor progression [2], [43]. Thus, certain upregulated microRNAs may prevent aggressive tumor growth through repression of the target genes that are responsible for tumor cell communication and extracellular matrix formation and may in part play a protective role in avoiding aggressive behavior of ULMs. Conversely, loss of *miR-200* shown to modulate growth as well as the UtLM morphology (Figure 4C,D), may lead to upregulation of genes (some of them convergent targets of lost *miR-15/16*) that contribute to the progression of ULM tumorigenesis.

Key components of the TGF-β and of MET signaling pathways are downmodulated in ULMs (Figure 5B and Table 4), both having a role in cell growth control, tumor progression, and oncogenic effects associated with invasive growth and extracellular matrix remodeling [44], [46]. Despite the downregulation of established TGF-β targets CTGF and THBS1 in ULMs (see Figure 5C, listing significant genes from EMT down category in Fig 5B), future studies will be required to determine whether microRNAs participate in TGF-β-mediated regulation of the profibrogenic extracellular matrix deposition in ULMs.

In conclusion, our global correlation analysis of gene product levels and microRNA expression provide a comprehensive and biologically meaningful insight into tumorigenesis of ULMs. This is documented by the relationship between the five most highly overexpressed microRNAs and a downregulated group of target genes that are related to cellular structure, extracellular matrix and cell proliferation, a signature that can be related to the slow growth and non aggressive behavior of uterine ULMs.

## Supporting Information

Figure S1 MicroRNA and mRNA expression profiles in five large uterine leiomyomas. microRNA and mRNA expression profiles in five large uterine leiomyomas from black women (Table 1). A. Unsupervised hierarchical clustering (HCL) of 206 human microRNAs in 5 large ULMs (>10 cm) normalized to the matched myometria is shown. B. HCL illustrating a concordance of total of 2674 significantly dysregulated genes between 5 ULMs from this study (left panel) and the NCBI GEO GSE593 data set (right panel), normalized to matched myometria. yellow = upregulation; blue = downregulation.
(0.69 MB JPG)

Figure S2 Regions of common loss in ULMs detected by array CGH. A, B. Genome browser mapping of the regions of common loss of genomic material as detected by a CGH in this study. Patient IDs are shown as yellow rectangles. Cancer-related microRNAs of interest in the deleted regions are listed under the chromosomes and highlighted by red arrowheads. C. Detailed genetic summary of the common regions of loss.
(0.60 MB JPG)

Figure S3 TMA immunohistochemical analysis of selected proteins in ULMs. Immunohistochemical analysis of selected proteins in 36 ULMs. A. Photomicrograph illustrating tissue microarray (TMA) sections with hematoxylin and eosin (H&E) stain (upper panel) and immunostaining of ER (bottom panel). Triplicate tissue cores from controls (matched myometrium) and tumors (ULMs) are indicated on the right. B. Differential expression of the selected target gene products is shown as mean and standard error of measurements (bars and t-bars, respectively). The net change for each gene product was calculated based on relative immunoreactivity in ULMs against matched myometrium. Red = gain in protein levels; Blue = reduction of protein levels.
(0.61 MB JPG)

Table S1 Primers and PCR results for the predicted target genes of let-7 and miR-200s.
(0.03 MB DOC)

Table S2 Antibodies used in this study.
(0.03 MB DOC)

Table S3 Predicted target genes and pathways downstream of miR-200s amd miR-15/16.
(0.81 MB DOC)
