# Supplementary figures and images for: Profiling and Functional Analyses of MicroRNAs and Their Target Gene Products in Human Uterine Leiomyomas
Source: PLoS One. 2010 Aug 24;5(8):e12362. doi: 10.1371/journal.pone.0012362 (PMC2927438; doi:10.1371/journal.pone.0012362)

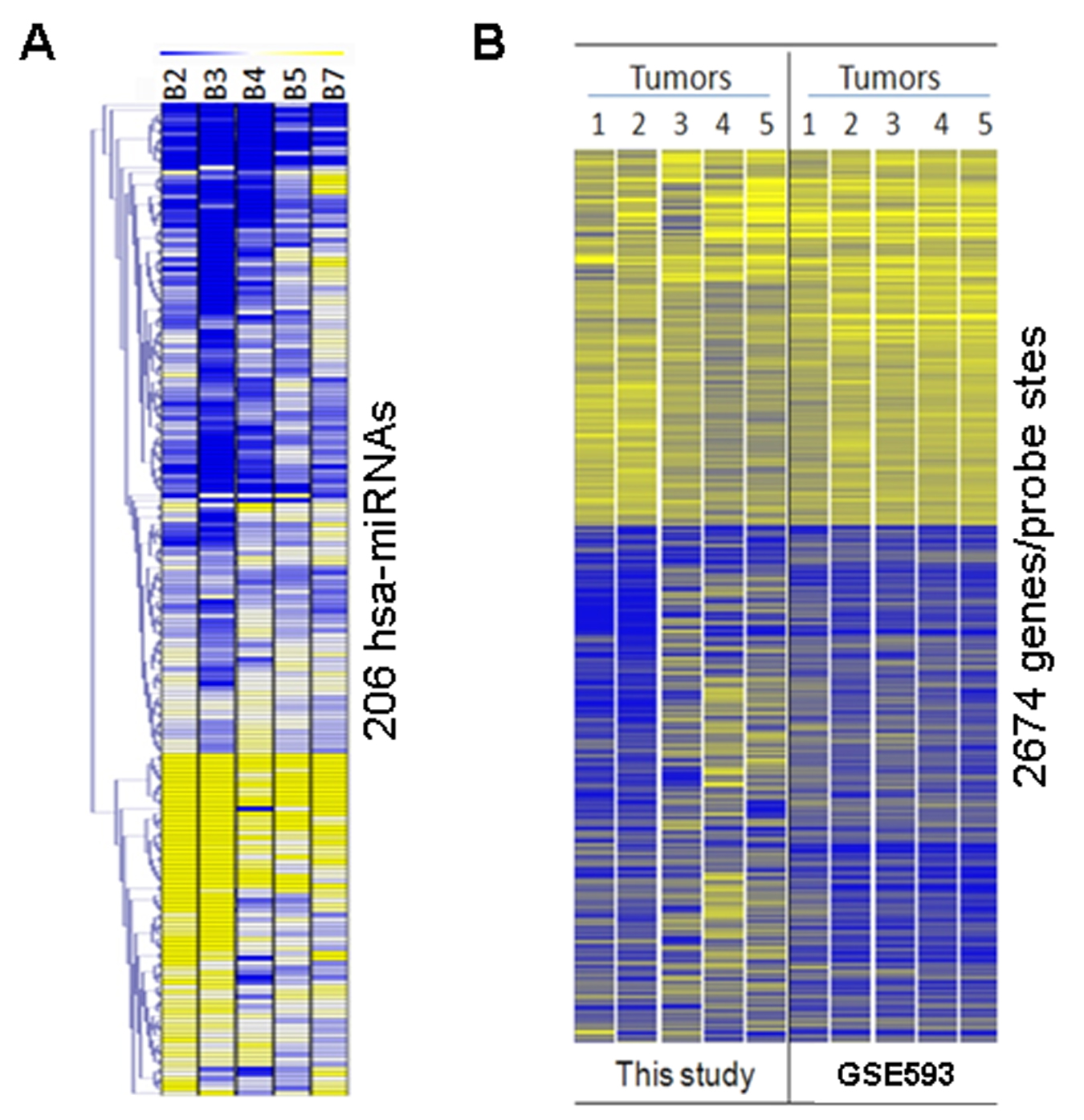

Supplement: Figure S1 — MicroRNA and mRNA expression profiles in five large uterine leiomyomas. microRNA and mRNA expression profiles in five large uterine leiomyomas from black women (Table 1). A. Unsupervised hierarchical clustering (HCL) of 206 human microRNAs in 5 large ULMs (>10 cm) normalized to the matched myometria is shown. B. HCL illustrating a concordance of total of 2674 significantly dysregulated genes between 5 ULMs from this study (left panel) and the NCBI GEO GSE593 data set (right panel), normalized to matched myometria. yellow = upregulation; blue = downregulation. (0.69 MB JPG) [file pone.0012362.s001.jpg]

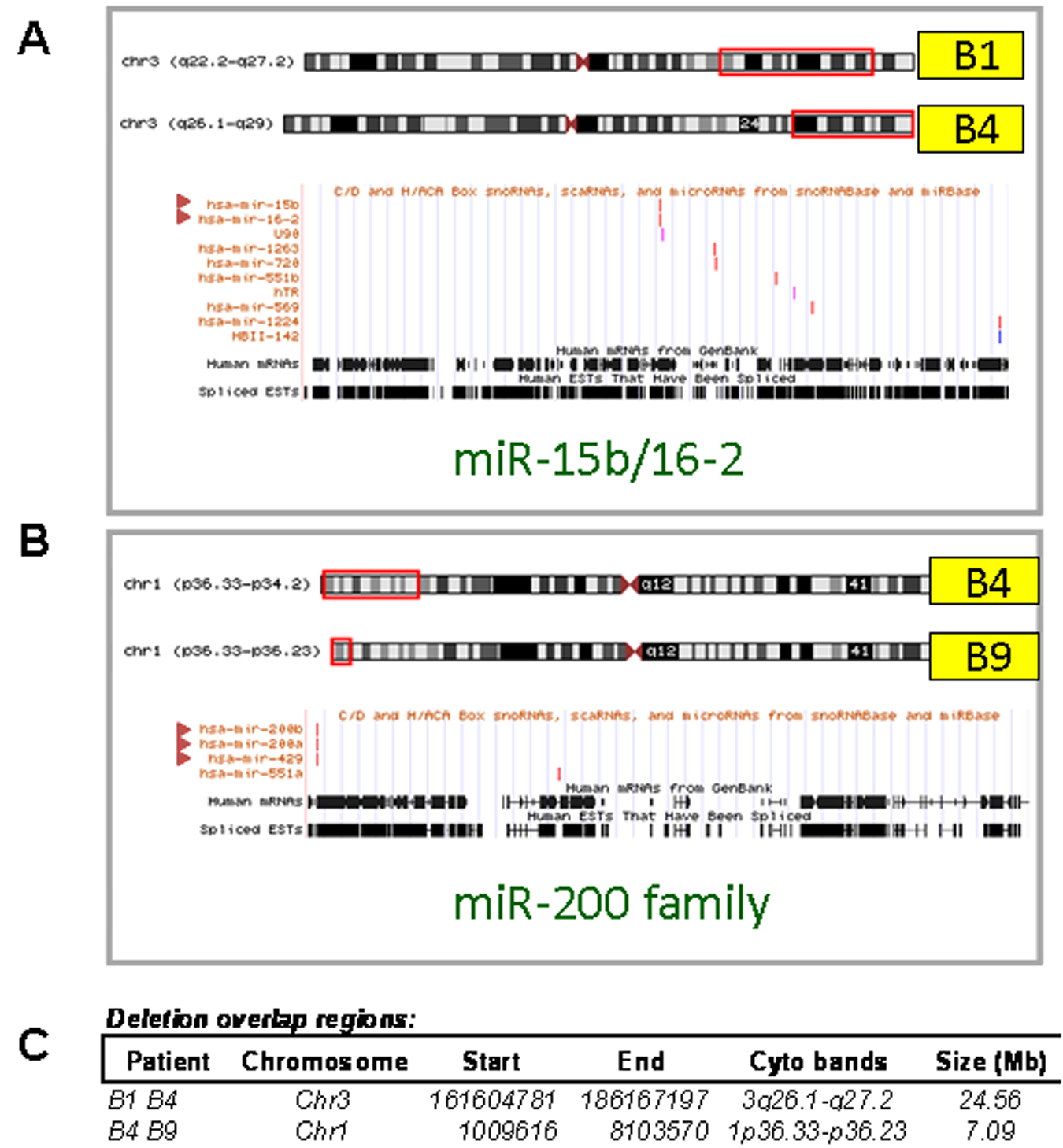

Supplement: Figure S2 — Regions of common loss in ULMs detected by array CGH. A, B. Genome browser mapping of the regions of common loss of genomic material as detected by a CGH in this study. Patient IDs are shown as yellow rectangles. Cancer-related microRNAs of interest in the deleted regions are listed under the chromosomes and highlighted by red arrowheads. C. Detailed genetic summary of the common regions of loss. (0.60 MB JPG) [file pone.0012362.s002.jpg]

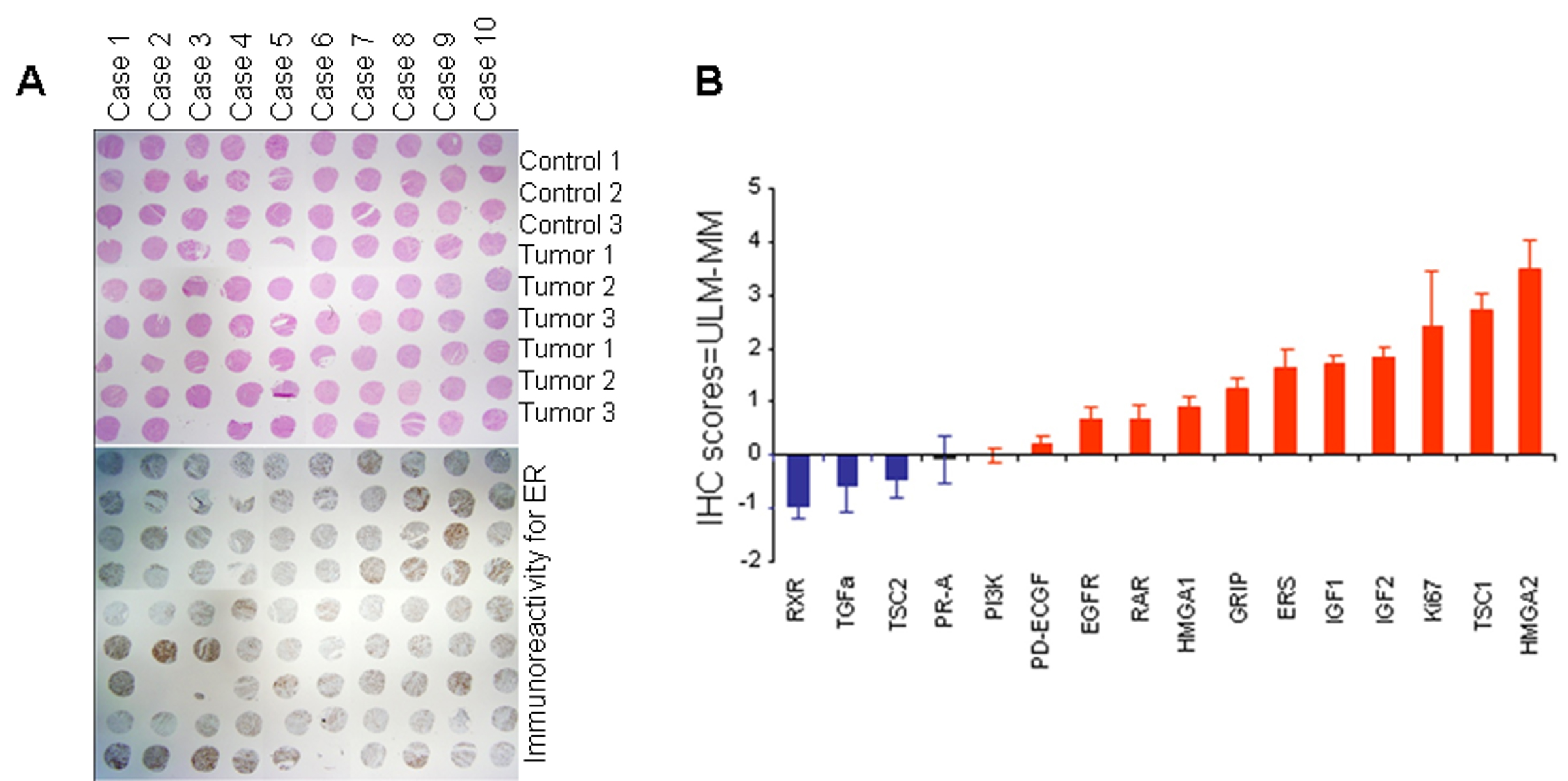

Supplement: Figure S3 — TMA immunohistochemical analysis of selected proteins in ULMs. Immunohistochemical analysis of selected proteins in 36 ULMs. A. Photomicrograph illustrating tissue microarray (TMA) sections with hematoxylin and eosin (H&E) stain (upper panel) and immunostaining of ER (bottom panel). Triplicate tissue cores from controls (matched myometrium) and tumors (ULMs) are indicated on the right. B. Differential expression of the selected target gene products is shown as mean and standard error of measurements (bars and t-bars, respectively). The net change for each gene product was calculated based on relative immunoreactivity in ULMs against matched myometrium. Red = gain in protein levels; Blue = reduction of protein levels. (0.61 MB JPG) [file pone.0012362.s003.jpg]
