# Supplementary material for: Profiling and Functional Analyses of MicroRNAs and Their Target Gene Products in Human Uterine Leiomyomas
Source: PLoS One. 2010 Aug 24;5(8):e12362. doi: 10.1371/journal.pone.0012362 (PMC2927438; doi:10.1371/journal.pone.0012362)
Supplement: Table S1 — Primers and PCR results for the predicted target genes of let-7 and miR-200s. (0.03 MB DOC) [file pone.0012362.s004.doc]

**Table S1** Primers and PCR results for the predicted target genes of let-7 and miR-200

Gene PicTar Fold Forward Primer Reverse Primer Amplicon

Symbol score Change (5’---3’) (5’---3’) length (bp)

*Let-7s* predicted targets

*HMGA2* 33 0.12 ggaagcagcagcaagaacc cctcttcggcagactcttgtga 228

*PPP1R12B* 14.44 0.28 aaggaacgacgaagaggcac cctctcccgtttctccatctc 413

*TRIB1* 10.05 -2.48 actgccaccagtcagccatc gagtaggtcccagtggtgttgag 200

*TRIB2* 7.45 0.70 gagttgtcgtctataaggtccgc cacataggctttggtctcaccc 309

*BTG2* 7.37 -1.25 gcgtgagcgagcagagg ggagactgccatcacgtagttc 386

*STARD13* 7.36 1.15 gtgtgcgagcagtggtgatg cacactctctgccacactcg 280

*ANGPTL2* 3.04 -1.10 gcctcatgcaggtgtggtg actcgctctcaggttccagg 272

*DUSP1* 2.72 -1.39 gacaaccacaaggcagacatc gttcgtggagtggacaggg 376

*DKK3* 2.19 -1.18 cttctggacctcatcacctgg tcttcagtcaggctcctctcc 250

*HMGA1* 2.07 - atgagtgagtcgagctcgaag agaaggaagctgctcctcca 333

*MiR-200s* predicted targets

*CYP1B1* 3.57 1.32 aactgtccatcaggtgaggt gttcgtggagtggacaggg 490

*ATXN1* 3.91 0.27 gacctcggtggagcttggtttac tgctgctcagccttgtgt 402

*CTBP2** 0.46 gtgatgattcctcctccacc tggacaggactatgaatcgg 291

*TNPO1** 0.49 tcgaggacggtggctcagca ggtacgtgtctggcggctgg 409

*TUBB* 3.51 0.32 gctgtaccaggcactgcggg ggctcgaactgcaaccgcct 145

* Two complementary sites by Target Scan 4.2 database.
