# Supplementary material for: Profiling and Functional Analyses of MicroRNAs and Their Target Gene Products in Human Uterine Leiomyomas
Source: PLoS One. 2010 Aug 24;5(8):e12362. doi: 10.1371/journal.pone.0012362 (PMC2927438; doi:10.1371/journal.pone.0012362)
Supplement: Table S2 — Antibodies used in this study. (0.03 MB DOC) [file pone.0012362.s005.doc]

**Table S2**  Antibodies used in this study

No. Markers Vendor/Source Titer

1 EGFR Zymed 1:20

2 ERα Ventana Medical Systems Neat

3 GRIP1 Affinity BioReagents 1:100

4 Hamartin Mizuguchi M et al (1997) 1:500

5 HMGA1 Santa Cruz 1:200

6 HMGA2 Santa Cruz 1:200

7 IGF1 Neo Markers 1:40

8 IGF-2 Santa Cruz 1:40

9 Ki-67 Ventana Medical Systems Neat

10 PD-ECGF Neo Markers 1:100

11 PI3K Santa Cruz 1:200

12 PR-A Ventana Medical Systems Neat

13 RARα Santa Cruz 1:100

14 RXRα Santa Cruz 1:50

15 TGF-α Neo Markers 1:50

16 TSC2 Mizuguchi M et al (1997) 1:700
