# Supplementary material for: Profiling and Functional Analyses of MicroRNAs and Their Target Gene Products in Human Uterine Leiomyomas
Source: PLoS One. 2010 Aug 24;5(8):e12362. doi: 10.1371/journal.pone.0012362 (PMC2927438; doi:10.1371/journal.pone.0012362)
Supplement: Table S3 — Predicted target genes and pathways downstream of miR-200s amd miR-15/16. (0.81 MB DOC) [file pone.0012362.s006.doc]

Footnote: Predicted target genes and pathways downstream of miR-200s amd miR-15/16. The pathway analyses based on predicted and correlated target genes of miR-200 and miR-15/16 was performed by NIH DAVID program (see Materials and Methods). Category - source database (Geneo ontology or KEGG). Term - description and ID of identified pathway. Count - number of genes found in each Term. P-value - significance of enrichment of the submitted gene list per Term. Genes - gene symbols mapping on each Term.
